# Derivatization procedure of estradiol with a combination of MPDNP-F and 4-dimethylaminopyridine to generate product ion containing estradiol-skeleton for reliable determination of its serum/plasma concentrations by LC/ESI-MS/MS

**DOI:** 10.1007/s00216-023-05069-9

**Published:** 2023-12-12

**Authors:** Honoka Kaneko, Hiroki Matsuoka, Takayuki Ishige, Hironori Kobayashi, Tatsuya Higashi

**Affiliations:** 1 Faculty of Pharmaceutical Sciences, Tokyo University of Science, Yamazaki, Noda, 2641 Chiba Japan; 2 Division of Laboratory Medicine, Chiba University Hospital, 1-8-1 Inohana, Chuo, Chiba 260-8677 Japan; 3 Clinical Laboratory Division, Shimane University Hospital, 89-1, Enya-cho, Izumo, Shimane 693-8501 Japan

**Keywords:** Derivatization, Estradiol, Skeleton-containing product ion, LC/ESI-MS/MS, Serum/plasma

## Abstract

The quantification of serum/plasma estradiol (E_2_) is useful for the diagnosis, pathological analysis, and monitoring of the therapeutic efficacy of estrogen-dependent diseases. In this study, an improved derivatization method using 1-(2,4-dinitro-5-fluorophenyl)-4,4-dimethylpiperazinium iodide (MPDNP-F) was developed and combined with liquid chromatography/electrospray ionization-tandem mass spectrometry (LC/ESI-MS/MS) for the sensitive and specific quantification of the serum/plasma E_2_. In the new method, the reaction time was reduced to 15 min from 90 min (two-step reaction in the previous method) by the direct reaction of MPDNP-F with E_2_ at 60°C in the presence of 4-dimethylaminopyridine (DMAP). DMAP served as the organic catalyst and had a less negative effect on the LC/ESI-MS/MS instrument compared to the non-volatile inorganic salt (NaHCO_3_), which was used in the previous method. The collision-induced dissociation of the molecular cation ([M]^+^) of the resulting derivative provided a product ion containing the E_2_-skeleton ([M–NO_2_–H]^+^), which significantly enhanced the assay sensitivity and specificity; compared to the dansyl chloride derivatization, which is the currently most-used derivatization procedure for the LC/ESI-MS/MS assays of E_2_, the MPDNP-F derivatization had significantly fewer interfering peaks and a clear and flat baseline in the serum sample analysis. The MPDNP-F derivatization–LC/ESI-MS/MS method enabled the precise and accurate quantification of E_2_ even at a 5.0 pg/mL concentration (lower limit of quantification) with a small sample volume (100 μL of serum/plasma) and had a tolerance for the matrix effect. This method was also proven to serve as a more sensitive and specific alternative to the clinically used chemiluminescence enzyme immunoassay.

**Graphical Abstract:**

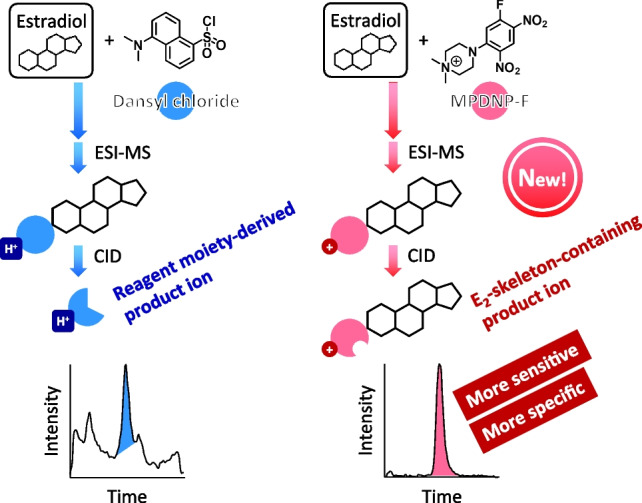

**Supplementary Information:**

The online version contains supplementary material available at 10.1007/s00216-023-05069-9.

## Introduction

The measurement of estradiol (E_2_), the most biologically active and clinically important estrogen, in the circulation is useful for the diagnosis, pathological analysis, and monitoring of the therapeutic efficacy of estrogen-dependent diseases, including gonadal dysfunction and breast cancer [1–3]. Although various immunoassays have been conventionally used for the quantification of E_2_ in routine clinical practice, it is now widely accepted that these assays have several important drawbacks, *i.e.*, a poor specificity due to cross-reactivity with other endogenous steroids, non-specific interactions with interfering substances, and poor agreement among the results obtained by different assay kits [1–3]. As an alternative method, liquid chromatography/electrospray ionization-tandem mass spectrometry (LC/ESI-MS/MS) has been used for the E_2_ quantification due to its high specificity. However, E_2_ has a rather low ESI efficiency in both the positive- and negative-ion modes due to its low proton affinity and weak acidic property. E_2_ also shows a poor fragmentation behavior during MS/MS, which is unfavorable for use of the selective reaction monitoring (SRM) mode. For these reasons as well as its very low blood concentration, derivatization has been often employed in the analysis of E_2_ in clinical samples for increasing its ESI-MS/MS detectability [3–5].

**Fig. 1:**
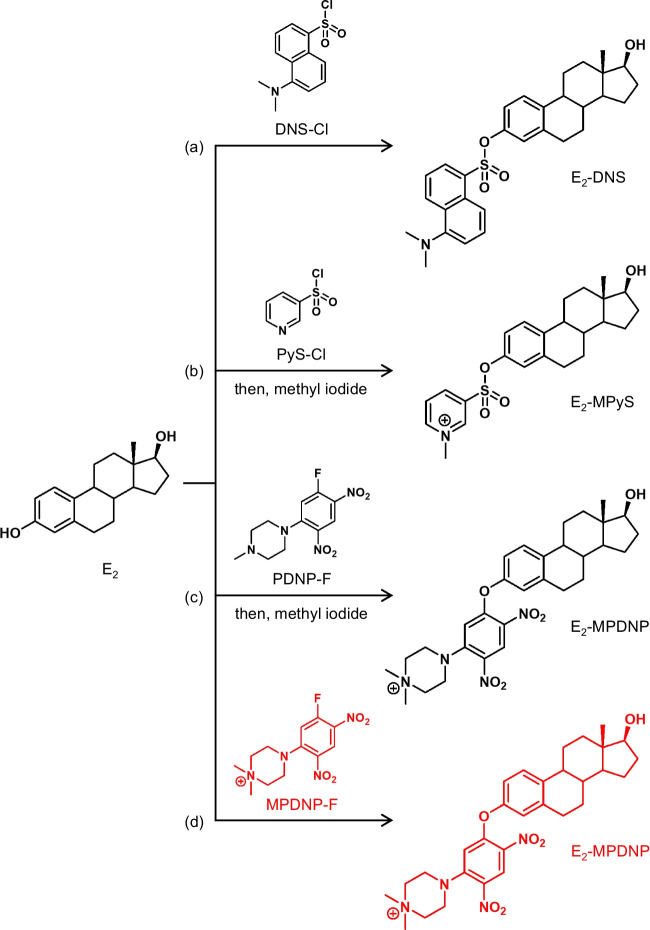
Derivatization reaction schemes of E_2_ with various reagents

**Fig. 2:**
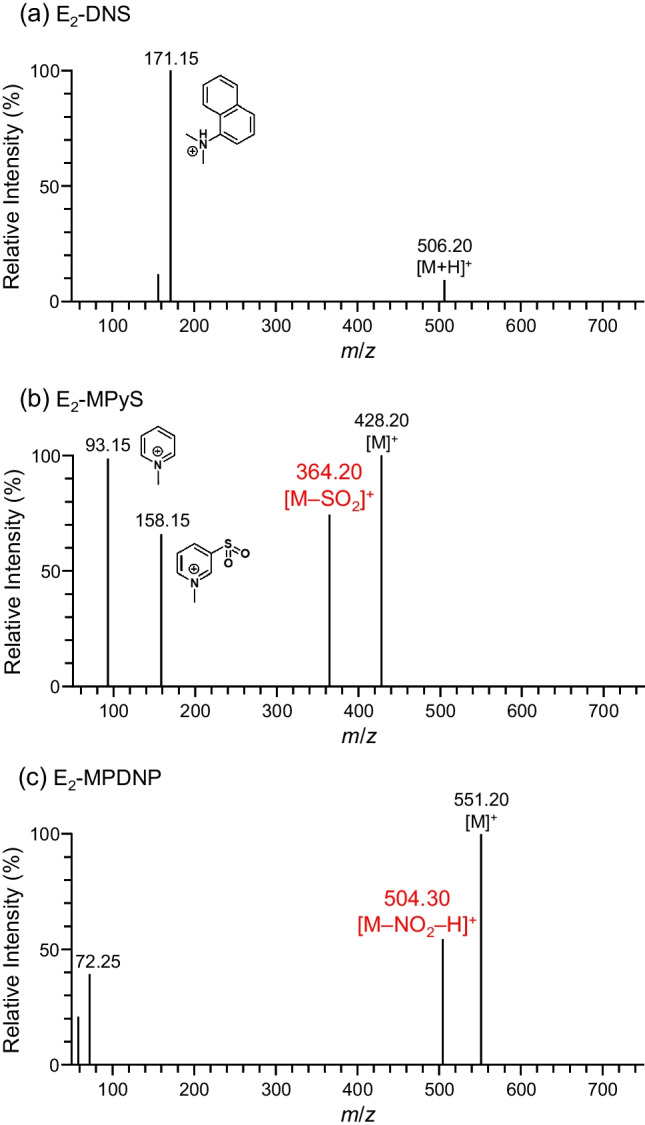
Product ion spectra of (**a**) E_2_-DNS, (**b**) E_2_-MPyS, and (**c**) E_2_-MPDNP

The dansyl chloride (DNS-Cl) derivatization (Fig. 1a) is now the most-used derivatization procedure for the LC/ESI-MS/MS assays of E_2_ [4–7]. By this derivatization, a tertiary amino group, which is readily protonated during ESI-MS using an acidic LC mobile phase, is introduced into E_2_, and the resulting derivative gives an intense product ion derived from the *N*,*N*-dimethylaminonaphthalene (DN) moiety (reagent-derived moiety) during MS/MS (Fig. 2a). Therefore, the detectability of the dansylated E_2_ is significantly greater than that of the intact E_2_ in ESI-MS/MS. However, the DNS-Cl derivatization has a major drawback; the protonated DN ([DN+H]^+^, *m*/*z* 171) is almost the only product ion of the dansylated E_2_ and this ion is also formed from the coexisting isobars of E_2_ if they are dansylated. Accordingly, a relatively high background noise and some interfering peaks often appear in the chromatograms of the biological samples even if the SRM is employed. Some different reagents including 2-fluoro-1-methylpyridine tosylate [8] and 3-bromomethyl-1-propyphenazone [9] also provide only the product ions composed of the reagent-derived moieties, and therefore, have a similar drawback to DNS-Cl.

The production of a specific product ion containing the E_2_-skeleton surely leads to the more sensitive and specific quantification of trace E_2_ in blood samples. As the reagents to fit this concept, pyridine-3-sulfonyl chloride (PyS-Cl) [6, 10] has been reported. The derivative with this reagent provided a product ion containing the E_2_-skeleton, *i.e.*, [M+H–SO_2_]^+^, from its protonated molecule ([M+H]^+^), and accordingly, was sensitively and specifically detected by ESI-MS/MS. Additional methylation after the PyS-Cl derivatization yielded a derivative possessing a permanently charged moiety (*N*-methylpyridium moiety) and also provided a product ion containing the E_2_-skeleton, *i.e.*, [M–SO_2_]^+^, from its molecular cation ([M]^+^) (Figs. 1b and 2b) [11]. This two-step derivatization consequently improved the sensitivity for detecting E_2_ although more effort and time were required. Thus, the PyS-Cl-based derivatization provided a derivative showing favorable ESI-MS/MS behavior unlike the derivatization using other sulfonyl chlorides, such as DNS-Cl and the recently developed analogues, 3-methyl-8-quinolinesulfonyl chloride (MQS-Cl) [12] and 4-acetylaminobenzenesulfonyl chloride [13]. For comparison, E_2_ derivatized with 1,2-dimethylimidazole-5-sulfonyl chloride [14] was also sensitively quantified by LC/atmospheric pressure photoionization-MS/MS with the SRM of [M+H]^+^ → [M+H–SO_2_]^+^, but this SRM transition was not applicable in ESI-MS/MS [10]. We have also developed 1-(2,4-dinitro-5-fluorophenyl)-4-methylpiperazine (PDNP-F; formerly named as PPZ) as the derivatization reagent for the sensitive analysis of estrogens by LC/ESI-MS/MS [15]. In the procedure using PDNP-F, E_2_ was first reacted with this reagent in the presence of NaHCO_3_ (base catalyst) at 60°C for 1 h, and then the resulting derivative was further reacted with methyl iodide at 60°C for 30 min (Fig. 1c). The obtained quaternized derivative gave the intense [M]^+^ in the positive ESI-MS, and furthermore, the product ion containing the E_2_-skeleton ([M–NO_2_–H]^+^) was generated by MS/MS (Fig. 2c). Due to these excellent characteristics, the PDNP-F derivatization was successfully used to quantify trace amounts of E_2_ in biological samples [16–18]. However, this derivatization procedure required two reaction steps, which are laborious and time-consuming. To overcome this drawback, we also synthesized the methylated PDNP-F (MPDNP-F; formerly named as MPPZ), *i.e.*, 1-(2,4-dinitro-5-fluorophenyl)-4,4-dimethylpiperazinium iodide, but achieved only a poor result in the derivatization of E_2_ when using NaHCO_3_ as the catalyst [15]. The one-step and rapid derivatization procedure using MPDNP-F for E_2_ would be beneficial for its sensitive and specific quantification in biological samples. Moreover, all the current derivatization procedures using DNS-Cl, PyS-Cl, and PDNP-F employ NaHCO_3_ or NaHCO_3_/Na_2_CO_3_ buffer as the base catalysts; these non-volatile inorganic salts are not friendly to an LC/MS/MS instrument even though a diversion valve is usually used to prevent these salts from entering the LC/MS/MS. Thus, the improved procedure using MPDNP-F is better without the non-volatile inorganic salt.

Based on this background information, the primary objective of this study was to develop the rapid one-step MPDNP-F derivatization procedure for E_2_ without the use of a non-volatile inorganic salt (Fig. 1d). To demonstrate the advantages of the developed procedure, it was compared to the DNS-Cl derivatization or PyS-Cl derivatization plus methylation in terms of sensitivity and specificity. Moreover, a sensitive and specific LC/ESI-MS/MS assay using the MPDNP-F derivatization was developed and validated for quantifying E_2_ in the order of pg/mL in serum/plasma samples.

## Experimental

### Chemicals and reagents

E_2_ and ^2^H_4_-E_2_ (internal standard, IS) were purchased from the Tokyo Chemical Industry (Tokyo, Japan) and Kanto Chemical (Tokyo), respectively, and dissolved in acetonitrile to prepare the 100 μg/mL stock solutions. The E_2_ working solutions of 50, 100, 200, 500, 1000, 2000, and 5000 pg/mL were prepared by subsequential dilutions of the stock solution with acetonitrile. The working solution of IS in acetonitrile (1000 pg/mL) was prepared and used. DNS-Cl, quinuclidine (QN), *N*,*N*-diisopropylethylamine (DIPEA), and methyl iodide were obtained from the Tokyo Chemical Industry. NaHCO_3_ and Na_2_CO_3_ were products of Kanto Chemical. PyS-Cl, 4-dimethylaminopyridine (DMAP), and triethylamine (TEA) were obtained from the FUJIFILM Wako Pure Chemical Corporation (Osaka, Japan). MPDNP-F and PDNP-F were synthesized in our laboratory by known methods [15]. MPDNP-F and PDNP-F could be used for the derivatization for at least 1 year when stored at −20°C. An Oasis^®^ HLB cartridge (30 mg adsorbent; Waters Corporation, Milford, MA, USA) was successively activated by acetonitrile (1 mL), methanol (1 mL), and water (1 mL) prior to loading pretreated samples for separation. A Strata™-X cartridge (60 mg adsorbent; Phenomenex, Torrance, CA, USA) was successively activated by ethyl acetate (2 mL), methanol (2 mL), and water (2 mL) prior to loading pretreated samples for separation. Ammonium formate, formic acid, and methanol used for the mobile phase were of LC/MS grade (FUJIFILM Wako Pure Chemical Corporation) and all other reagents and solvents were of analytical grade. Water was purified on a Puric-α system (Organo, Tokyo).

### Serum and plasma samples

Apparently healthy male subjects in their 20s to 40s (*n* = 15) and apparently healthy non-pregnant female subjects in their 20s to 40s with different phases of their menstrual cycle (*n* = 15) donated their blood with the full understanding of the purpose of this study and their written informed consents. The blood collection was performed at Chiba University (Chiba, Japan). The serum was separated by centrifugation after blood coagulation, and then stored at −30°C until used. Serum samples obtained from the female patients (15–74 years old) attending the Shimane University Hospital (Izumo, Japan) were also used in this study. These samples were first used for diagnostic purposes by measuring the E_2_ concentrations by chemiluminescence enzyme immunoassay (CLEIA) [AIA-PACK CL^®^ Estradiol kit and AIA^®^ CL-2400 instrument (Tosoh Corporation, Tokyo)] in the hospital, and the remaining frozen samples (stored at −30°C) were used for this study; the patients agreed the use of their serum samples in this study. The experimental procedures were approved by the Institutional Review Boards of Chiba University (No. 640), Shimane University Hospital (No. KS20230222-2) and Tokyo University of Science (No. 23012, Noda, Japan).

Human Serum Standard Reference Materials (BCR-576 and BCR-578) were purchased from Sigma-Aldrich Japan (Tokyo) for the method development and validation; these sera are certified to contain 31.50 ± 1.36 and 365.00 ± 19.04 pg/mL of E_2_, respectively. Several lots of FFP-LR Nisseki frozen plasma (the Japan Red Cross Service, Tokyo) containing 16.5–31.5 pg/mL of E_2_ were also used for the method development and validation.

### Pretreatment of serum/plasma sample

The serum/plasma sample (100 μL) was added to acetonitrile (200 μL) containing IS (10 pg) [mixture of acetonitrile (190 μL) and the IS solution (1000 pg/mL in acetonitrile, 10 μL)], and then vortex-mixed for 30 s for deproteinization. After the centrifugation (1000 × *g*, 20°C, 10 min), the supernatant was diluted with water (500 μL) in the reservoir of the Oasis^®^ HLB cartridge, and then loaded on the cartridge. After washing with water (1 mL) and methanol-water (1:1, v/v, 1 mL), E_2_ and IS were eluted with acetonitrile (1.5 mL). The solvent was evaporated under N_2_ at 40°C and the residue was subjected to derivatization.

### MPDNP-F derivatization (new one-step method using DMAP)

To the standard E_2_ or the pretreated serum/plasma sample, MPDNP-F in acetonitrile (2 mg/mL, 20 μL) and DMAP in acetonitrile (1 mg/mL, 20 μL) were added. The mixture was heated at 60°C for 15 min. The solvent was evaporated under N_2_ at 40°C, and then the residue was dissolved in the mobile phase (40 μL), one-quarter of which was injected into the LC/ESI-MS/MS.

### MPDNP-F derivatization (previous two-step method using NaHCO_3_)

PDNP-F in acetonitrile (2 mg/mL, 20 μL) and 1 M NaHCO_3_ (20 μL) were added to the standard E_2_, and then this mixture was heated at 60°C for 1 h [15]. The reaction mixture was diluted with methanol-water (1:1, v/v, 500 μL) and passed through a Strata™-X cartridge for desalting. After washing with water (2 mL), the PDNP-F-derivatized E_2_ was eluted with ethyl acetate (1 mL). After evaporation of the solvent, methyl iodide (100 μL) was added, and then the reaction mixture was left stand at 60°C for 30 min. The mixture was evaporated to dryness under N_2_ at 40°C, and then the residue was dissolved in the mobile phase (40 μL), one-quarter of which was injected into the LC/ESI-MS/MS.

### DNS-Cl derivatization using QN

To the standard E_2_ or the pretreated serum/plasma sample, DNS-Cl in acetonitrile (2 mg/mL, 20 μL) and QN in acetonitrile (1 mg/mL, 20 μL) were added. The mixture was heated at 60°C for 15 min. After the solvent was evaporated, the residue was dissolved in the mobile phase (40 μL), one-quarter of which was injected into the LC/ESI-MS/MS.

The conventional DNS-Cl derivatization procedure using the NaHCO_3_/Na_2_CO_3_ buffer is described in the Supplementary Information.

### PyS-Cl derivatization using QN followed by methylation

To the standard E_2_, PyS-Cl in acetonitrile (2 mg/mL, 20 μL) and QN in acetonitrile (1 mg/mL, 20 μL) were added. The mixture was heated at 60°C for 15 min. After evaporation of the solvent, 20% (v/v) methyl iodide in acetonitrile (500 μL) was added, and then the reaction mixture was left stand at 80°C for 30 min. The mixture was evaporated to dryness under N_2_ at 40°C, and then the residue was dissolved in the mobile phase (40 μL), one-quarter of which was injected into the LC/ESI-MS/MS.

The conventional PyS-Cl derivatization procedure using the NaHCO_3_/Na_2_CO_3_ buffer is described in the Supplementary Information.

### MPDNP-F derivatization rate

#### Derivatization rate for nanogram amount of E_2_

E_2_ (4.0 ng) was derivatized by the one-step MPDNP-F derivatization procedure and dissolved in the mobile phase (40 μL). One-quarter of this solution [equivalent to 1.0 ng of intact E_2_] was then subjected to LC/ESI-MS/MS under the conditions for detecting the intact E_2_. We determined if the peak of the intact E_2_ was detected or not [limit of detection (LOD) for the intact E_2_ was 20 pg]. If the E_2_ peak is not detected, it follows that the amount of E_2_ that remains underivatized is less than 20 pg of the origin amount (1.0 ng), indicating that the derivatization yield is quantitative (more than 98%).

#### Derivatization rate for picogram amount of E_2_

Different amounts (1.0 ng and 50 pg) of E_2_ were individually derivatized, and then dissolved in 800 and 40 μL of the mobile phase, respectively. Ten microliters of the respective solutions was subjected to LC/ESI-MS/MS. The peak areas were compared (*n* = 3); if the peak areas are almost equal, the derivatization rate of a picogram amount of E_2_ is almost identical to that of the nanogram amount of E_2_.

##### Influence of the serum/plasma components on the MPDNP-F derivatization

Sample A: the serum/plasma sample (100 μL) was pretreated as previously described. After the addition of IS (10 pg; 10 μL of the 1000 pg/mL solution), the sample was derivatized with MPDNP-F, dissolved in the mobile phase (40 μL), and subjected to LC/ESI-MS/MS (10 μL) (*n* = 5). Sample B: the serum/plasma sample (100 μL) was pretreated as previously described. IS (10 pg; 10 μL of the 1000 pg/mL solution) was separately derivatized, dissolved in the mobile phase (40 μL), and then added to the pretreated serum/plasma sample. The resulting sample was subjected to LC/ESI-MS/MS (10 μL) (*n* = 5). The influence of the serum/plasma components on the derivatization was evaluated by comparing the peak areas of the derivatized IS in samples A and B; if the peak areas are almost equal, the endogenous components are considered to have little influence on the derivatization.

##### Effect of derivatization on detection response

An appropriate amount of E_2_ (0.20–1.0 pg) was derivatized with MPDNP-F, DNS-Cl, or PyS-Cl plus methyl iodide, and the resulting derivatives were dissolved in the mobile phases (40 μL), and then one-quarter of which was subjected to LC/ESI-MS/MS. The intact E_2_ (80 pg) dissolved in the mobile phase (40 μL) was also analyzed. The amounts of the intact and derivatized E_2_ giving a signal-to-noise ratio (*S*/*N*) of 3 (LODs) were determined. The methanol ratio in the mobile phases was adjusted so that the retention times (*t*_R_s) of the intact and derivatized E_2_ were around 5 min (Table 1).

**Table 1:** Optimized LC/ESI-MS/MS conditions and *t*_R_s

Compound	Mobile phase^a^	*t*_R_ (min)	Q1 (V)	Q3 (V)	CE (eV)	SRM transition^b^
E_2_ (intact)	A	4.7	13	27	46	271.3 [M–H]^–^ → 145.2
E_2_-MPDNP	B (3:2)	5.2	−28	−32	43	551.2 [M]^+^ → 504.3 [M–NO_2_–H]^+^
IS-MPDNP	B (3:2)	5.1	−26	−34	44	555.4 [M]^+^ → 507.3 [M–NO_2_–^2^H]^+^
E_2_-DNS	B (5:1)	4.8	−22	−29	17	506.2 [M+H]^+^ → 171.1 [DN+H]^+^
E_2_-MPyS	B (1:1)	4.6	−12	−28	28	428.2 [M]^+^ → 364.2 [M–SO_2_]^+^

^a^A: methanol-water (8:5, v/v), B: mixture of methanol and 10 mM ammonium formate containing 0.1% (v/v) formic acid. The ratios of methanol and 10 mM ammonium formate are shown in parentheses

^b^Precursor ion → product ion (*m*/*z*). DN represents dimethylaminonaphthalene

##### LC/ESI-MS/MS

The LC/ESI-MS/MS instrument was comprised of a Shimadzu LCMS-8030^+^ triple quadrupole mass spectrometer and a Shimadzu LC-30AD chromatograph (Kyoto, Japan). A YMC-UltraHT Pro C18 (2.0 µm, 100 × 2.0 mm i.d., Kyoto) was used at the flow rate of 0.3 mL/min and at the temperature of 40°C. The derivatized and intact E_2_ were analyzed in the positive-ion and negative-ion modes, respectively. The MS/MS conditions common to all the compounds were as follows: interface voltage, 4.5 or −3.5 kV; detector voltage, 2.16 or −2.12 kV; nebulizer gas (N_2_) flow rate, 3 L/min; drying gas (N_2_) flow rate, 15 L/min; desolvation line temperature, 250°C; heat block temperature, 400°C; and collision gas (Ar), 230 kPa. The Q1 pre-rod bias voltage (Q1), Q3 pre-rod bias voltage (Q3), collision energy (CE), SRM transitions (precursor and product ions), and mobile phases (isocratic elution) for the respective compounds are described in Table 1. LabSolutions software (version 5.53 SP3, Shimadzu) was used for the system control and data processing.

##### Calibration curve

The serum/plasma was stirred with one-tenth the weight of activated charcoal for 15 h, and then centrifuged (2000 × *g*, 10 min). For the obtained supernatant, this procedure was repeated to completely remove E_2_; the resulting supernatant was used as the blank serum/plasma for constructing the calibration curves. This blank serum/plasma (100 μL) was added to acetonitrile (200 μL) containing E_2_ (0.50–50 pg) and IS (10 pg) [mixture of acetonitrile (180 μL), the E_2_ solution (50, 100, 200, 500, 1000, 2000, or 5000 pg/mL in acetonitrile, 10 μL), and the IS solution (1000 pg/mL in acetonitrile, 10 μL)]; these corresponded to the calibration samples with the E_2_ concentration of 5.0, 10, 20, 50, 100, 200, or 500 pg/mL and the IS concentration of 100 pg/mL. The resulting sample was pretreated and derivatized as previously described. The calibration curve was constructed by plotting the peak area ratios (E_2_-MPDNP/IS-MPDNP, *y*) versus the E_2_ concentrations (pg/mL, *x*). The linear regression was fitted with a weighting factor of 1/*x*. The linearity was evaluated based on the determination coefficient (*r*^2^).

##### Precision and accuracy

The intra- and inter-assay precision and accuracy were assessed by five repetitive measurements of the following four samples on 1 day and over 5 days, respectively. The relative standard deviations (RSDs, %) and relative errors (REs, %) of the measured values were used for evaluating the assay precision and accuracy, respectively. The acceptance criteria were met if the RSDs and REs were ≤ 20% and within ± 20%, respectively, for the lower limit of quantification (LLOQ) sample, and ≤ 15% and within ± 15%, respectively, for the quality control (QC) samples.

The LLOQ sample (nominal E_2_ concentration, 5.00 pg/mL) was prepared by mixing BCR-576 (nominal E_2_ concentration, 31.05 pg/mL) and the blank serum at a ratio of 1:5.21. The low concentration QC sample (QC1) was the undiluted BCR-576. The medium concentration QC sample (QC2, nominal E_2_ concentration, 97.84 pg/mL) was prepared by mixing BCR-576 and BCR-578 (nominal E_2_ concentration, 365.00 pg/mL) at a ratio of 4:1. The high concentration QC sample (QC3) was the undiluted BCR-578.

##### Matrix effect

The peak areas (absolute values) of the MPDNP-F-derivatized E_2_ and IS (E_2_- and IS-MPDNP) in the matrix samples were divided by those in the standard samples to determine the matrix factors (MFs). Furthermore, the ratios of the derivatized E_2_/IS in the matrix samples were divided by those in the standard samples to determine the IS-normalized MFs (ISMFs). The MFs and ISMFs were expressed as a percentage.

Standard sample: a mixture of E_2_ and IS (1.0 ng each) was derivatized with MPDNP-F and dissolved in the mobile phase (1.0 mL, *n* = 5). Matrix sample: the serum/plasma sample was pretreated as previously described. To the obtained residue, one-hundredth the quantity of the above standard sample (10 μL) and the mobile phase (30 μL) were added to prepare the matrix sample (*n* = 5).

## Results and discussion

### Optimized conditions for the one-step MPDNP-F derivatization

Our initial effort was directed toward finding an organic base suitable for the one-step MPDNP-F derivatization for E_2_. We evaluated four tertiary amines, *i.e.*, DMAP, QN, DIPEA, and TEA. In parallel to this, the derivatization solvent (methanol, ethanol, and acetonitrile), temperature (room temperature, 60°C, and 80°C), and time (15 and 30 min) were also systematically tested. Among the bases, the largest peak of E_2_-MPDNP was obtained when DMAP was used. QN was slightly less effective than DMAP, and DIPEA and TEA could not compare with DMAP. Although the derivatization reaction proceeded even at room temperature, it took more than an hour to complete the reaction. On the other hand, the heating at 60°C completed the reaction within 15 min; a longer time was ineffective for increasing the desired derivative. The heating at 80°C reduced the yield of the derivative contrary to our expectation. In regard to this phenomenon, we examined the stability of MPDNP-F on heating and found that this reagent was stable at 60°C, but partially decomposed at 80°C; the 1,1-dimethylpiperazine dissociated from the reagent. This was considered a major cause of the decreased yield of the derivatized product by the heating at 80°C. Acetonitrile was overwhelmingly superior to methanol as the derivatization solvent, and ethanol could not be used for the derivatization because MPDNP-F did not dissolve in this solvent. Reproducible results were obtained when more than 40 µg of MPDNP-F was used. The amounts of the derivatized product did not significantly vary within the DMAP amount range of 20–40 µg; therefore, 20 µg was selected as the optimum amount of DMAP. Thus, the derivatization conditions were optimized as described in the “Experimental” section.

The peak area of the derivative obtained by the new one-step method using DMAP was compared to that obtained by the previous two-step method using NaHCO_3_ [15]; the former was somewhat greater (about 20% increase) than the latter. The total reaction time was reduced to 15 min by the one-step derivatization method, whereas the previous two-step method required a total of 90 min [15]. Furthermore, the previous two-step method required the desalting and solvent-evaporation processes between the two reactions [15], whereas the one-step method did not need them; not only the reaction time but also the processing time and labor for the derivatization were drastically reduced in the one-step method. Thus, the one-step method using DMAP was far superior to the previous two-step method in several respects.

The derivatization rate was calculated based on the experiments described in the “Experimental” section. The derivatization rate was inferred to be quantitative (nearly 100%) when the nanogram amount of E_2_ was subjected to the derivatization, because E_2_ that remained underivatized was not detected (the minimum detectable amount of the intact E_2_ was 20 pg). Furthermore, the amount of the derivative produced in the picogram-scale reaction was consistent with that of the derivative produced in the nanogram-scale reaction and adequately diluted; this result indicated that the derivatization quantitatively proceeded even for a picogram amount of E_2_. The influence of the serum/plasma components for the derivatization will be discussed in a later section.

### Applicability of DMAP and QN as alternatives to the inorganic base catalysts in other derivatization

As already described, we found that DMAP and QN were workable as the base catalysts for the MPDNP-F derivatization of E_2_. Therefore, applicability of these amines as alternatives to the inorganic base catalysts (NaHCO_3_/Na_2_CO_3_) in the other derivatization was examined. In the DNS-Cl derivatization, the peak area remained largely unchanged by using QN instead of the NaHCO_3_/Na_2_CO_3_ buffer [6, 7] (both reactions proceeded at 60°C for 15 min), whereas DMAP gave an unsatisfactory result because the product amount by using DMAP was about 70% of that by using the NaHCO_3_ buffer. Although Tang and Guengerich reported that the combination of DMAP and DIPEA strongly promoted the dansylation for various compounds [19], we revealed that QN is useful as the organic base catalyst for the DNS-Cl derivatization of E_2_. For the PyS-Cl derivatization of E_2_, the NaHCO_3_/Na_2_CO_3_ buffer has also been used as the base catalyst [6, 10]. The replacement of this buffer by QN (in acetonitrile) caused no problem for this derivatization, whereas the use of DMAP significantly lowered the yield (40% compared to NaHCO_3_/Na_2_CO_3_ buffer or QN). Based on these results, QN was used as the catalyst in the DNS-Cl and PyS-Cl derivatization in the subsequent experiment.

### Effect of derivatization on the detection response

To evaluate the effects of each derivatization for increasing the detectability of E_2_, the LODs of the resulting derivatives were compared (Table 2). Because the peak intensity was dependent on the *t*_R_, the derivatives were eluted at around 5 min by adjusting the methanol ratio in the mobile phase (Table 1). It was highly noticeable that the LOD of E_2_-MPDNP prepared by the one-step method (0.18 fmol) was one four-hundredth of that of the intact E_2_ (74 fmol). Furthermore, E_2_-MPDNP showed the highest sensitivity among the tested derivatives; E_2_-MPDNP was detected two times more sensitively than E_2_-DNS. The peak intensity of E_2_-DNS was significantly higher than that of E_2_-MPDNP, but the noise was also significantly greater, which led to a lower *S*/*N* value. This result was thought to be due to the low specificity of the reagent moiety-derived product ion ([DN+H]^+^) of the DNS-Cl derivative. E_2_-MPyS provided the product ions containing the E_2_-skeleton ([M–SO_2_]^+^), but its detectability (LOD 0.92 fmol) could not compare to that of E_2_-MPDNP due to the low intensity of this product ion.

**Table 2:** LODs of intact E_2_ and its derivatives

Compound	LOD^a^ (fmol)	Increasing sensitivity^b^
E_2_ (intact)	74	1
E_2_-MPDNP	0.18	400
E_2_-DNS	0.37	200
E_2_-MPyS	0.92	80

^a^The injection amounts producing a peak with an *S*/*N* of 3

^b^The detection sensitivity of the intact E_2_ is taken as 1

### Pretreatment procedure

The serum/plasma samples were purified by protein precipitation and solid-phase extraction (SPE), and then derivatized. The recovery rates of E_2_ and IS during the SPE were determined using the blank serum/plasma samples spiked with the known amounts of E_2_ and IS (the detailed procedure is described in the Supplementary Information). As shown in Table 3, E_2_ and IS were satisfactorily and reproducibly recovered from the serum/plasma samples.

**Table 3:** Recovery rate during SPE and matrix effect

	Serum			Plasma		
	Recovery rate (%)	MF (%)	ISMF (%)	Recovery rate (%)	MF (%)	ISMF (%)
E_2_	88.1 ± 1.8	86.4 ± 4.3	100.7 ± 2.1	87.0 ± 1.6	88.1 ± 2.8	100.2 ± 0.6
IS	87.8 ± 0.7	85.8 ± 4.1	–	86.9 ± 1.7	88.0 ± 3.0	–

Results are shown in mean ± SD (five different samples)

**Fig. 3:**
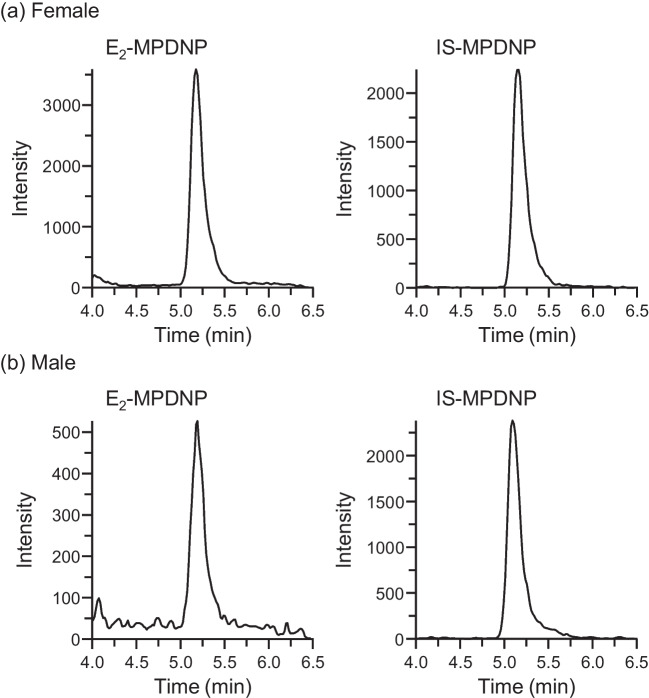
SRM chromatograms of E_2_ and IS in serum samples collected from (**a**) female and (**b**) male subjects as the derivatives with MPDNP-F. The measured concentrations were (a) 112.5 and (b) 14.5 pg/mL, respectively

Typical chromatograms of the serum samples obtained from the apparently healthy female and male subjects are shown in Fig. 3, in which the peaks corresponding to E_2_- and IS-MPDNP were clearly observed without any interference from the matrix components. Even for the male sample, which contained a low level of E_2_ compared to the female sample, the peak of the derivatized E_2_ was observed with a sufficient *S*/*N* (Fig. 3b). The influence of the serum/plasma components for the derivatization was evaluated by the experiment described in the “Experimental” section. The peak area ratio of the IS derivatized in the presence and absence of the serum components was 99.0 ± 1.3% [mean ± standard deviation (SD), *n* = 5]. For the plasma sample, the ratio was 100.4 ± 2.3%. Thus, the serum/plasma components had no effect on the extent of the derivatization reaction due to the appropriate pretreatment procedure.

### Calibration curve

The reproducibility and linearity of the calibration curves were evaluated by preparing five curves each for serum and plasma using five different blank samples. The regression formulas were *y* = (0.01402 ± 0.00045)*x* + (0.01437 ± 0.00190) for the serum and *y* = (0.01380 ± 0.00022)*x* + (0.02392 ± 0.00526) for the plasma (mean ± SD were given for the slopes and *y*-intercepts); the curves were reproducibly constructed for both matrices as the RSDs of the slope were 3.2 and 1.6%, respectively. All the curves showed a good linearity in the range of 5.0–500 pg/mL as the *r*^2^ values were ≥ 0.998. Although the LLOQ (*i.e.*, lowest calibration point) of 5.0 pg/mL might not be sufficient for quantifying the serum/plasma E_2_ of some postmenopausal female subjects (< 10 pg/mL [2, 3]), especially for those receiving aromatase inhibitor therapy (< 5 pg/mL [1]), this was largely due to the use of the Shimadzu LCMS-8030^+^ mass spectrometer, which was the low-end model of Shimadzu’s LC/MS/MS instrument lineup (discontinued model) and had been used for over 10 years in our laboratories. The LLOQ of the MPDNP-F derivatization method will be improved by using the latest high-performance mass spectrometer because it has been demonstrated that 2.0 pg/mL of E_2_ could be quantified after conversion of it to E_2_-MPDNP (prepared by the previous two-step method) using the AB Sciex QTRAP 6500^+^ mass spectrometer [17]. The LLOQ may become lower with a larger sample volume. However, our method was optimized and validated for a 100-μL serum/plasma sample; for a large increase of the sample volume, some major modifications will be required in the pretreatment of samples and derivatization conditions. In a preliminary experiment, some 200-μL serum/plasma samples were analyzed by our current method. As a result, nearly the same quantitative values on the E_2_ concentrations were obtained with about twice peak intensities and no significant increase of background noise, compared to when the corresponding 100-μL samples were used. This result indicated the possibility that our current method is adaptable enough for a 200-μL serum/plasma sample and the LLOQ can be consequently lowered to 2.5 pg/mL. This point will be validated in our future study. Table 4 shows the reported LLOQs of some recent derivatization–LC/ESI-MS/MS assays of E_2_ in human serum/plasma. Although the method employing the PyS-Cl derivatization followed by methylation had a remarkable LLOQ (0.5 pg/mL) [11], as previously noted, this procedure gave a disappointing result for increasing the sensitivity in our replication study; we were unsure about the cause of this discrepancy.

**Table 4:** LLOQs of derivatization–LC/ESI-MS/MS assays of E_2_ in human serum/plasma

Derivatization reagent	Sample volume (μL)	Instrument	LLOQ (pg/mL)	Reference
MPDNP-F	100 (serum/plasma)	LCMS-8030^+^ (Shimadzu)	5.0	This study
DNS-Cl	100 (serum)	QTRAP 6500^+^ (AB Sciex)	2.0	[6]
DNS-Cl	200 (serum)	QTRAP 6500 (AB Sciex)	2.0	[7]
PyS-Cl	100 (serum)	QTRAP 6500^+^ (AB Sciex)	5.0	[6]
PyS-Cl + CH_3_I	100 (serum)	TSQ Vantage (Thermo Fisher Scientific)	0.5	[11]
MQS-Cl	100 (serum)	LCMS-8050 (Shimadzu)	10	[13]
PDNP-F + CH_3_I	500 (plasma)	QTRAP 6500^+^ (AB Sciex)	2.0	[17]

### Precision and accuracy

Good results were demonstrated for the assay precision and accuracy at any concentrations (LLOQ, low, medium, and high QCs) (Table 5). The RSDs of the intra- and inter-assay measurements (*n* = 5) were ≤ 6.7% and ≤ 3.0%, respectively. The intra- and inter-assay REs ranged from −2.2 to 0.2% and from −2.7 to −0.7%, respectively.

**Table 5:** Assay precision and accuracy

	LLOQ (5.00 pg/mL)	QC1 (31.05 pg/mL)	QC2 (97.84 pg/mL)	QC3 (365.00 pg/mL)
Intra-assay				
Measured concentration (pg/mL)^a^	4.89 ± 0.33	30.78 ± 0.23	98.02 ± 2.82	361.67 ± 7.85
RSD (%)	6.7	0.8	2.9	2.2
RE (%)	−2.2	−0.9	0.2	−0.9
Inter-assay				
Measured concentration (pg/mL)^a^	4.95 ± 0.15	30.84 ± 0.19	97.08 ± 2.23	354.97 ± 6.19
RSD (%)	3.0	0.6	2.3	1.7
RE (%)	−1.0	−0.7	−0.8	−2.7

^a^Mean ± SD (*n* = 5)

### Matrix effect

As the MFs of E_2_ and IS were in the upper 80s (%) for both the serum and plasma samples, a mild ion suppression was observed for these matrices (Table 3). However, the ISMFs were very close to 100% for both matrices. Thus, the use of IS satisfactorily compensated for the matrix effect in our method.

### Stability

Many studies demonstrated that E_2_ is stable in serum/plasma under the frozen condition for at least a month [6, 9], at 4°C for at least a week [6, 7], and at 25°C for a few days [6]; the stability of E_2_ in the serum/plasma samples was not examined in this study. After three cycles of freezing (−30°C) and thawing (*ca*. 25°C), negligibly small changes were observed in the E_2_ concentrations for the serum (98.9 ± 1.6%, mean ± SD, five different samples) and plasma samples (101.1 ± 1.9%).

The derivatized E_2_ in the pretreated serum/plasma samples was stable in the autosampler (20°C) for at least 24 h; the measured concentrations after 24 h of storage were 99.7 ± 2.5 and 99.9 ± 2.8% (mean ± SD, five different samples) of those just after the preparation for the serum and plasma samples, respectively.

### Advantage of MPDNP-F derivatization versus DNS-Cl derivatization in the serum E_2_ quantification

**Fig. 4:**
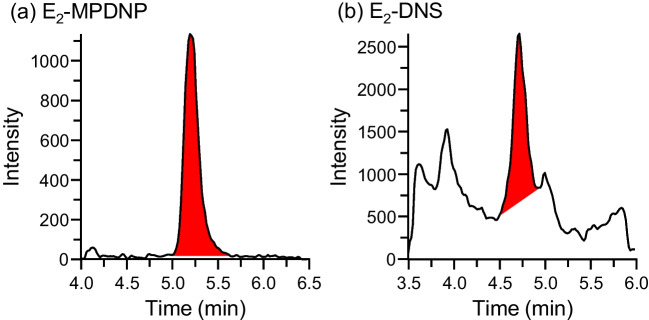
SRM chromatograms of E_2_ in same serum samples as the derivatives with (**a**) MPDNP-F and (**b**) DNS-Cl. The E_2_ concentration determined by the method using MPDNP-F was 29.0 pg/mL

To demonstrate the advantage of the MPDNP-F derivatization versus the DNS-Cl derivatization, which is now the most-used derivatization procedure for the LC/ESI-MS/MS assays of E_2_, the SRM chromatograms of the same serum sample after the MPDNP-F or DNS-Cl derivatization are shown in Fig. 4. As described in the “Introduction” section, E_2_-DNS almost provides only the reagent moiety-derived product ion, [DN+H]^+^, during the MS/MS (Fig. 2a). As a result, a high background noise (winding baseline) and many interfering peaks appeared when E_2_ was detected as E_2_-DNS (Fig. 4b). On the other hand, negligible interfering peaks derived from the serum components were observed and a clear and flat baseline was obtained when the MPDNP-F derivatization was employed (Fig. 4a). This specific detection became possible due to the formation of the E_2_-skeleton-containing product ion ([M–NO_2_–H]^+^). The absolute peak intensity was greater in E_2_-DNS, but the detectability was significantly greater in E_2_-MPDNP due to very low background noise (*i.e.*, increased *S*/*N*), indicating that the MPDNP-F derivatization is better suited for biological sample analyses.

### Applicability of MPDNP-F derivatization

To demonstrate the applicability of the one-step MPDNP-F derivatization procedure, the serum E_2_ concentrations of apparently healthy subjects (male and female subjects in their 20s to 40s, *n* = 15 each) were measured by LC/ESI-MS/MS combined with this derivatization. Although clinical importance of the quantification of the serum/plasma E_2_ in the male is under debate, the male samples were measured as a substitute for the low E_2_-containing female samples for the applicability evaluation. The E_2_ concentrations of the male and female subjects were 15.7 ± 3.8 pg/mL (range, 11.6–23.2 pg/mL) and 134.4 ± 70.7 pg/mL (34.4–291.5 pg/mL), respectively; these concentrations were consistent with the previously reported concentrations [2, 3].

**Fig. 5:**
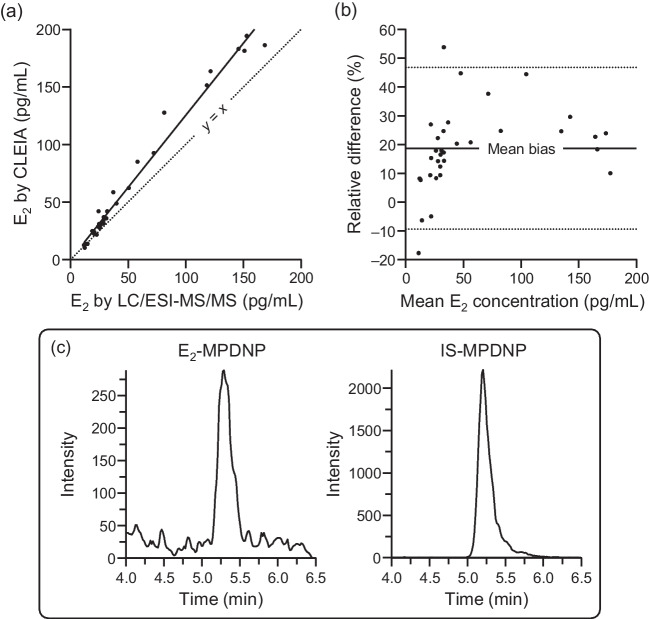
**a** Scatter plot to compare the measured E_2_ concentrations by CLEIA and developed LC/ESI-MS/MS. **b** Bland-Altman plot of the mean E_2_ concentration and the relative difference between CLEIA and LC/ESI-MS/MS. The relative difference was calculated as follows: [(E_2_ concentration by CLEIA) − (E_2_ concentration by LC/ESI-MS/MS)] / mean E_2_ concentration × 100 (%). The dashed lines represent the 95% limits of agreement. (**c**) SRM chromatograms of the serum samples whose E_2_ concentration was below the LLOQ of CLEIA. The E_2_ concentration was determined to be 8.7 pg/mL by the developed LC/ESI-MS/MS

The E_2_ concentrations measured by the MPDNP-F derivatization–LC/ESI-MS/MS were compared with those by the commercially available CLEIA kit (AIA-PACK CL^®^ Estradiol). In this experiment, the female serum samples whose E_2_ concentrations had been determined to be within the range of 10–200 pg/mL by the CLEIA (*n* = 33) were used. According to the manufacturer’s instructions, this CLEIA shows a very low cross-reactivity with other endogenous estrogens: 17α-E_2_ (0.01%), estrone (0.37%), estriol (0.11%), E_2_-3-sulfate-17-glucuronide (not detectable), E_2_-17-glucuronide (0.01%), E_2_-3-glucuronide-17-sulfate (not detectable), and E_2_-3-sulfate (0.01%). However, the linear regression line was (E_2_ concentration by CLEIA, *y*) = 1.246 × (E_2_ concentration by LC/ESI-MS/MS, *x*) + 0.412 with a correlation coefficient (*r*) of 0.991 (Fig. 5a). The Bland-Altman plot showed that the E_2_ concentrations measured by the CLEIA were 18.7% (ranged from −17.7 to 53.0%) higher than those determined by LC/ESI-MS/MS (Fig. 5b). These results indicated a significant positive bias in the E_2_ quantification by the CLEIA. Although we could not provide a plausible reason for this, non-specific interactions with some serum substances during the antigen-antibody reaction or enzyme reaction were suspected. Furthermore, the CLEIA could not provide quantitative values for the samples whose E_2_ concentrations were below 10 pg/mL, because the CLEIA’s LLOQ was 10 pg/mL. Some of such samples were analyzed by the developed LC/ESI-MS/MS method and chromatograms obtained from one such sample are shown in Fig. 5c, in which a quantifiable peak of the derivatized E_2_ was observed. Based on these results, it can be safely said that our LC/ESI-MS/MS method is superior to the commercial CLEIA in sensitivity as well as specificity for clinical sample analysis.

## Conclusion

We developed an improved derivatization method using MPDNP-F for the sensitive and specific quantification of the serum/plasma E_2_ by LC/ESI-MS/MS. In the new method, the derivatization was quantitatively completed by the one-step reaction, which reduced the total reaction time from 90 min (previous two-step method) to 15 min, and DMAP was used as the organic catalyst, which has a less negative effect on the LC/MS/MS instrument compared to the non-volatile inorganic salt used in the previous method and made the desalting process unnecessary. The resulting derivative, E_2_-MPDNP, provided a specific product ion containing the E_2_-skeleton ([M–NO_2_–H]^+^) during the MS/MS. Therefore, the MPDNP-F derivatization more significantly enhanced the assay sensitivity and specificity than the often-utilized derivatization with DNS-Cl, especially in the real sample (serum/plasma) analysis. The MPDNP-F derivatization followed by LC/ESI-MS/MS enabled the precise and accurate quantification of E_2_ even at the 5.0 pg/mL concentration (LLOQ) with a small sample volume (100 μL of serum/plasma) and had a tolerance for the matrix effect. This method was applied to the serum sample analysis and proven to serve as a more specific alternative to the clinically used CLEIA.

### Supplementary Information

Below is the link to the electronic supplementary material.

Supplementary file1 (DOCX 28.1 KB)

## Data Availability

Data will be made available upon request.

## Declarations

### Source of biological material

Deidentifed human serum samples were collected at the Chiba University Hospital and Shimane University Hospital. Human Serum Standard Reference Materials (BCR-576 and BCR-578) and pooled human plasma FFP-LR (Nisseki frozen plasma) were purchased from Sigma-Aldrich Japan and Japan Red Cross Service, respectively.
