# Supplementary material for: Derivatization procedure of estradiol with a combination of MPDNP-F and 4-dimethylaminopyridine to generate product ion containing estradiol-skeleton for reliable determination of its serum/plasma concentrations by LC/ESI-MS/MS
Source: Anal Bioanal Chem. 2023 Dec 12;416(2):597–608. doi: 10.1007/s00216-023-05069-9 (PMC10761386; doi:10.1007/s00216-023-05069-9)
Supplement: Supplementary file 1 — Supplementary file1 (DOCX 28.1 KB) [file 216_2023_5069_MOESM1_ESM.docx]

Supplementary Information

**Derivatization procedure of estradiol with a combination of** **MPDNP-F and 4-dimethylaminopyridine to generate product ion containing estradiol-skeleton for reliable determination of its serum/plasma concentrations by LC/ESI-MS/MS**

**Honoka Kaneko^1^ ∙ Hiroki Matsuoka^1^ ∙ Takayuki Ishige^2^ ∙ Hironori Kobayashi^3^ ∙ Tatsuya Higashi^1^**

^1^ Faculty of Pharmaceutical Sciences, Tokyo University of Science, 2641 Yamazaki, Noda, Chiba 278-8510, Japan

^2^ Division of Laboratory Medicine, Chiba University Hospital, 1‒8‒1 Inohana, Chuo, Chiba 260‒8677, Japan

^3^ Clinical Laboratory Division, Shimane University Hospital, 89-1, Enya-cho, Izumo, Shimane 693-8501, Japan

Corresponding author

Tatsuya Higashi

higashi@rs.tus.ac.jp (T. Higashi)

**DNS-Cl derivatization (conventional method using** **NaHCO_3_/Na_2_CO_3_ buffer)**

To the standard E_2_, DNS-Cl in acetonitrile (2 mg/mL, 20 μL) and 50 mM NaHCO_3_/Na_2_CO_3_ buffer (pH 10.5, 20 μL) were added. The resulting mixture was heated at 60ºC for 15 min. The reaction mixture was diluted with ethyl acetate (100 μL), then washed with water (100 μL, two times) for desalting. The organic layer was transferred to another tube, then the solvent was evaporated.

**PyS-Cl derivatization (conventional method using NaHCO_3_/Na_2_CO_3_ buffer)**

To the standard E_2_, PyS-Cl in acetone (1 mg/mL, 80 μL) and 100 mM NaHCO_3_/Na_2_CO_3_ (pH 10.5, 80 μL) were added. The resulting mixture was heated at 60ºC for 15 min. The reaction mixture was diluted with ethyl acetate (100 μL), then washed with water (100 μL, two times) for desalting. The organic layer was transferred to another tube, then the solvent was evaporated.

**Absolute recovery rates during solid phase extraction**

Sample I: The blank serum/plasma (100 μL, *n* = 5) was added to acetonitrile (200 μL) containing E_2_ (10 pg) [mixture of acetonitrile (190 μL) and the E_2_ solution (1000 pg/mL in acetonitrile, 10 μL)] and pretreated. After the addition of IS (10 pg) [the IS solution (1000 pg/mL in acetonitrile, 10 μL)], the sample was derivatized with MPDNP-F, dissolved in the mobile phase (40 μL) and subjected to LC/ESI-MS/MS (10 μL).

Sample II: The blank serum/plasma (100 μL, *n* = 5) was added to acetonitrile (200 μL) and pretreated. After the addition of E_2_ (10 pg; 10 μL of the 1000 pg/mL solution) and IS (10 pg; 10 μL of the 1000 pg/mL solution), the sample was derivatized with MPDNP-F, dissolved in the mobile phase (40 μL) and subjected to LC/ESI-MS/MS (10 μL).

Sample III: The blank serum/plasma (100 μL, *n* = 5) was added to acetonitrile (200 μL) containing IS (10 pg) [mixture of acetonitrile (190 μL) and the IS solution (1000 pg/mL in acetonitrile, 10 μL)] and pretreated. After the addition of E_2_ (10 pg; 10 μL of the 1000 pg/mL solution), the sample was derivatized with MPDNP-F, dissolved in the mobile phase (40 μL) and subjected to LC/ESI-MS/MS (10 μL).

The absolute recovery rate of E_2_ was calculated based on the following formula; recovery (%) = [peak area ratio (E_2_/IS) in sample I / peak area ratio in sample II] × 100. The recovery rate of IS was calculated based on the following formula; recovery (%) = [peak area ratio (IS/E_2_) in sample III / peak area ratio in sample II] × 100.
